# Peripheral Administration of NMU Promotes White Adipose Tissue Beiging and Improves Glucose Tolerance

**DOI:** 10.1155/2021/6142096

**Published:** 2021-08-02

**Authors:** Yue Yuan, Hongdong Wang, Jielei He, Haixiang Sun, Dalong Zhu, Yan Bi

**Affiliations:** Department of Endocrinology, Affiliated Drum Tower Hospital, Medical School of Nanjing University, Nanjing, China

## Abstract

**Purpose:**

Targeting white adipose tissue (WAT) beiging has been proposed as an effective way to increase thermogenesis and improve glucose metabolism. Neuromedin U (NMU) is a neuropeptide that could increase energy expenditure, while its effects on WAT beiging and glucose homeostasis remain to be investigated.

**Methods:**

Male C57BL/6 mice were fed with high fat diet (HFD) to induce obesity and hyperglycemia and then treated with chronic subcutaneous injection of NMU. Body weight and food intake were recorded daily. After 14 days of injection, intraperitoneal glucose tolerance tests and 18F-fluorodeoxyglucose micro-positron emission tomography/computed tomography (18F-FDG micro-PET/CT) scans were conducted. Subcutaneous WAT (sWAT) and interscapular brown adipose tissue were collected for the evaluation of adipocyte size, expression of uncoupling protein 1 (*Ucp1*), and other thermogenic-related genes. Stromal vascular fraction of subcutaneous WAT was extracted for the measurement of type 2 innate lymphocytes (ILC2s) proportions.

**Results:**

Glucose tolerance was markedly improved by peripherally administered NMU. Micro-PET/CT suggested that NMU promoted WAT beiging, which was further confirmed by haematoxylin and eosin (H&E) staining and immunohistochemistry. In diet-induced-obese (DIO) mice, NMU activated thermogenic-related genes in WAT. In addition, NMU stimulated ILC2s in the stromal vascular fraction of WAT.

**Conclusion:**

Taken together, our study indicates that peripheral administration of NMU is a potential therapeutic strategy for the promotion of WAT beiging and the improvement of impaired glucose tolerance.

## 1. Introduction

Over the past decades, the prevalence of obesity and diabetes increased rapidly, causing great social burden around the world [1–3]. As a major risk factor of type 2 diabetes mellitus, obesity is associated with the imbalance between energy intake and energy expenditure and is characterized with expanded adipose tissue (AT) mass in the body [4, 5]. Classically, AT is divided into three categories, white adipose tissue (WAT), beige adipose tissue, and brown adipose tissue (BAT). The primary function of WAT is energy storage, while beige and brown AT are sites for energy expenditure. BAT consists of multilocular adipocytes with increased numbers of mitochondria that express uncoupling protein 1 (UCP1). BAT dissipates energy through nonshivering thermogenesis, oxidizing fatty acids produced by triglyceride hydrolysis to generate heat. Beige AT refers to brown-like adipocytes sporadically distributed within WAT, which usually arises through the process called WAT beiging upon stimulation of cold, PPAR agonists, or exercise. When stimulated, beige adipocytes express UCP1 protein and exhibit thermogenic capacity at a comparable level to BAT [4, 6, 7]. In obese patients, WAT is excessively accumulated in the body, causing local immune abnormalities, tissue fibrosis, and AT secretome changes, which drive peripheral insulin resistance and contribute to the pathogenesis of type 2 diabetes mellitus [4]. Previous studies have demonstrated that WAT beiging is associated with reduced AT dysfunction and fibrosis. Beige AT is also related to improved glucose and lipid homeostasis [8–10]. However, effective ways to promote WAT beiging are still under investigation.

Neuromedin U (NMU) is a peptide widely distributed in different kinds of neurons (cholinergic, noncholinergic, and sensory) throughout the body. It is highly expressed in the gastrointestinal tract, as well as many other tissues, including AT [11, 12]. NMU is conserved across different mammalian species and is involved in multiple important physiological and pathophysiological processes, participating in the regulation of energy homeostasis [12]. In a Denmark population, homozygous carriers of NMU gene mutations had increased prevalence of overweight and obesity [13]. NMU^−/−^ mice were more susceptible to obesity and late-onset hyperglycemia [14]. In rodents, administration of NMU led to not only decreased food intake, lowered body weight, and improved glucose homeostasis, but also activation of BAT, featured by elevated expression of *Ucp1* in BAT, increased heat production, and body temperature [15, 16]. Moreover, *Ucp1* expression was decreased in BAT of NMU^−/−^ mice [14]. As a matter of fact, NMU and NMU receptor 1 (NMUR1) were found to express in WAT in human [17]. Nevertheless, whether NMU could promote WAT beiging and improve metabolic homeostasis remains unknown.

Recently, the neuro‐immune system crosstalk mediated by NMU-type 2 innate lymphocytes (ILC2s) was discovered. Peripherally injected NMU activated ILC2 cells in small intestine lamina propria and lung [18]. Intraperitoneal injection of an RNase glycoprotein extracted from the Helminth induced ILC2s accumulation in epididymal WAT, leading to weight loss and glucose tolerance improvements in obese mice [19]. In AT, ILC2s, upon activation, produced type 2 cytokines, stimulated eosinophils and type 2 (or M2) macrophages to promote WAT beiging and thermogenesis [20]. However, it remains to be clarified whether peripherally administrated NMU activates ILC2s in AT and facilitates WAT beiging.

In this study, we aimed to investigate the impact of NMU on glucose metabolism and thermogenic AT in diet-induced-obese (DIO) mice. After chronic subcutaneous injection of NMU, blood glucose levels, morphology of AT, and expression of genes related to thermogenesis in BAT and WAT were evaluated. NMU improved glucose tolerance, activated BAT, and promoted WAT beiging in DIO mice. Taken together, our study suggests that peripheral administration of NMU has potential metabolic benefits in promoting WAT beiging and ameliorating impaired glucose tolerance.

## 2. Methods

### 2.1. Animal Experiments

C57Bl/6 mice were purchased from Shanghai SLAC Laboratory Animal Co., Ltd, China. Mice were housed in a standard animal room of controlled environment (constant light-dark cycler, lights off at 20:00). Food and water were given ad libitum. 8-week-old mice were randomly divided into normal chow diet (NCD) or high fat diet (HFD) (D12492, Research Diets) groups and fed accordingly until the end of the research. After 6 weeks, mice in the HFD group were randomly divided into normal saline- (NS-) treated and NMU-treated (NMU23, Phoenix Pharmaceuticals) group (HFD + NS group, *n* = 10; HFD + NMU group, *n* = 10). NMU (8 *μ*g/day) or NS of the same volume was subcutaneously injected into the interscapular space daily for 14 days. The NCD group was also subcutaneously injected with NS of equal volume (NCD + NS group, *n* = 12). Body weight and food intake were recorded daily.

### 2.2. Intraperitoneal Glucose Tolerance Test (IpGTT)

IpGTT was performed in all three groups after 14 days of NMU or NS injection. Food was withdrawn 12 hours before the test. After measurement of fasting blood glucose using tail blood, mice were treated with 2 g/kg D-glucose solution (10%) by intraperitoneal injection. Blood glucose levels were measured at 15, 30, 60, and 120 min after glucose challenge.

### 2.3. 18F-FDG Micro-PET/CT

Micro-PET/CT was conducted on a Siemens Inveon PET/CT System in the Department of Nuclear Medicine of Nanjing First Hospital. After 18 hours of fasting and 4 hours of cold exposure (4°C), mice were kept in 30°C environment, with food and water given ad libitum. 30 minutes later, mice were intraperitoneally injected with 18F-FDG (4 *μ*Ci/g). After one hour of radiotracer uptake, mice were anesthetized with constant isoflurane inhalation and fixed in a stretched position for PET/CT scan. CT scanning at 80 kV and 500 *μ*A took 10 min, followed by 5 min of PET signal collection. Coregistration of the reconstructed PET and CT images and all image analysis were performed using the manufacturer's software.

### 2.4. Isolation of AT and SVF

After 14 days of NMU or NS injection, mice were anesthetized in chambers saturated with isoflurane and then sacrificed by cervical dislocation. For H&E staining and immunohistochemistry, interscapular BAT and inguinal subcutaneous WAT (sWAT) were dissociated and fixed for 24 h using fat fixative (Wuhan Sevicebio Technology Co., Ltd). For SVF preparation, BAT and sWAT were cut into small pieces (∼1 mm^3^) and digested with 0.1% type I collagenase (Sigma-Aldrich, USA) in 37°C water bath. During digestion, the fat pieces were kept in tubes and mixed every 2 min by turning the tubes upside down. After 40–45 min, the digestion process was terminated by adding equal volume of phosphate buffer saline (PBS). The specimens were centrifuged at 400x for 5 min, and the supernatants were then aspirated. The sediments were washed with PBS, filtered through a 100 *μ*m nylon mesh, and resuspended in red blood cell lysis buffer (GE, USA). After red blood cell lysis, the remaining SVF was washed again with PBS.

### 2.5. Histology and Immunofluorescence

BAT and sWAT in fixatives were sectioned after being paraffin-embedded. Sectioned tissues were stained with H&E for general morphological observation. Immunohistochemistry was conducted using *Ucp1* antibodies (BD, Bioscience, 1:100) and secondary antibodies (anti-rabbit, Alexa Fluor 488, Life Technologies Inc., Carlsbad, CA, USA, 1:100). Images were captured and digitalized with microscope (Olympus, Tokyo, Japan). *Ucp1* expression was quantified using NIH ImageJ software.

### 2.6. Quantitative Real-Time PCR Analysis (qPCR)

The total RNA was extracted from BAT and sWAT using trizol (Life Technologies, Thermo Fisher Scientific) following standard protocols. Complementary DNA was prepared from 1 mg RNA with the PrimerScript RT Master Mix (TAKARA, Japan) according to the manufacturer's instructions. Quantitative PCR reactions were run by LightCycler 480 Real-Time PCR System (Roche, Switzerland). Fold changes in gene expression were calculated as 2^−△△Ct^ with *β*-actin as the housekeeping gene. Sequences of the primers are listed in Supplementary Table 1.

### 2.7. Flow Cytometry

Mouse SVF samples were stained with the following antibodies: lineage (Lin) antibodies CD3, CD5, TCR*α/β*, CD19, CD56, CD11c, CD11b, and CD16 (BD, Bioscience). Other antibodies used for the characterization of ILC2s included L/D, CD45, and ST2 (BD, Bioscience). The stained cells were analyzed on an LSR II Fortessa flow cytometer (BD, Bioscience). ILC2s were identified as Lin^−^CD45^+^ST2^+^. Data were analyzed using FACSDiva software (BD Bioscience) and FlowJo software version 9.6.4 (Tree Star, Inc).

### 2.8. Statistical Analyses

All statistical analyses were performed with SPSS version 22.0 (SPSS Inc., USA). Differences were determined using ANOVA tests for normally distributed variables. Data were presented as mean ± SEM unless otherwise stated. *p* values <0.05 were considered statistically significant.

## 3. Results

### 3.1. Chronic Subcutaneous Injection of NMU Exerts Beneficial Effects on Glucose Tolerance

**Figure 1:**
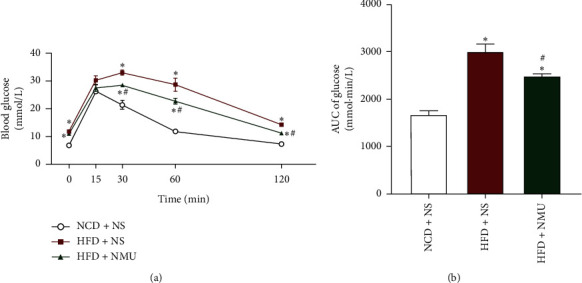
Chronic subcutaneous administration of NMU improves glucose tolerance in DIO mice. (a) Blood glucose levels and (b) area under the curve of glucose levels of ipGTT after NMU administration (*n* = 5−6). ^*∗*^*p* < 0.05 (compared to NCD + NS group). ^#^*p* < 0.05 (compared to HFD + NS group).

Chronic subcutaneous administration of NMU significantly lowered blood glucose levels 30, 60, and 120 minutes after glucose injection (Figure 1(a)). Area under the curve of blood glucose in ipGTT was also significantly decreased in DIO mice treated with NMU (Figure 1(b)). NMU injection did not induce significant changes in body weight (Supplementary Figure 1(a)) or food intake (Supplementary Figure 1(b)) as compared to NS in DIO mice. Elevated fasting blood glucose (FBG) in DIO mice was not affected by NMU (Supplementary Figure 1(c)). These results suggest that NMU could improve glucose tolerance when energy intake and body weight remain steady.

### 3.2. NMU Activates BAT and Enhances Thermogenesis

**Figure 2:**
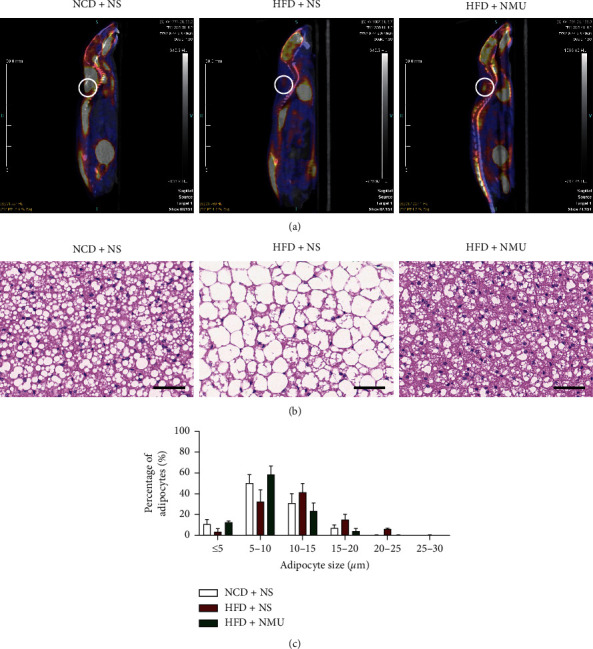
NMU increases FDG-uptake in BAT and restores BAT morphology. (a) Sagittal view of micro-PET/CT image after cold stimulation and 18F-FDG injection. (b) Representative H&E staining of interscapular BAT. Scale bar, 50 *μ*m. (c) Quantification of adipocytes of different diameters in interscapular BAT (*n* = 3). ^*∗*^*p* < 0.05 (compared to the NCD + NS group). ^#^*p* < 0.05 (compared to the HFD + NS group).

**Figure 3:**
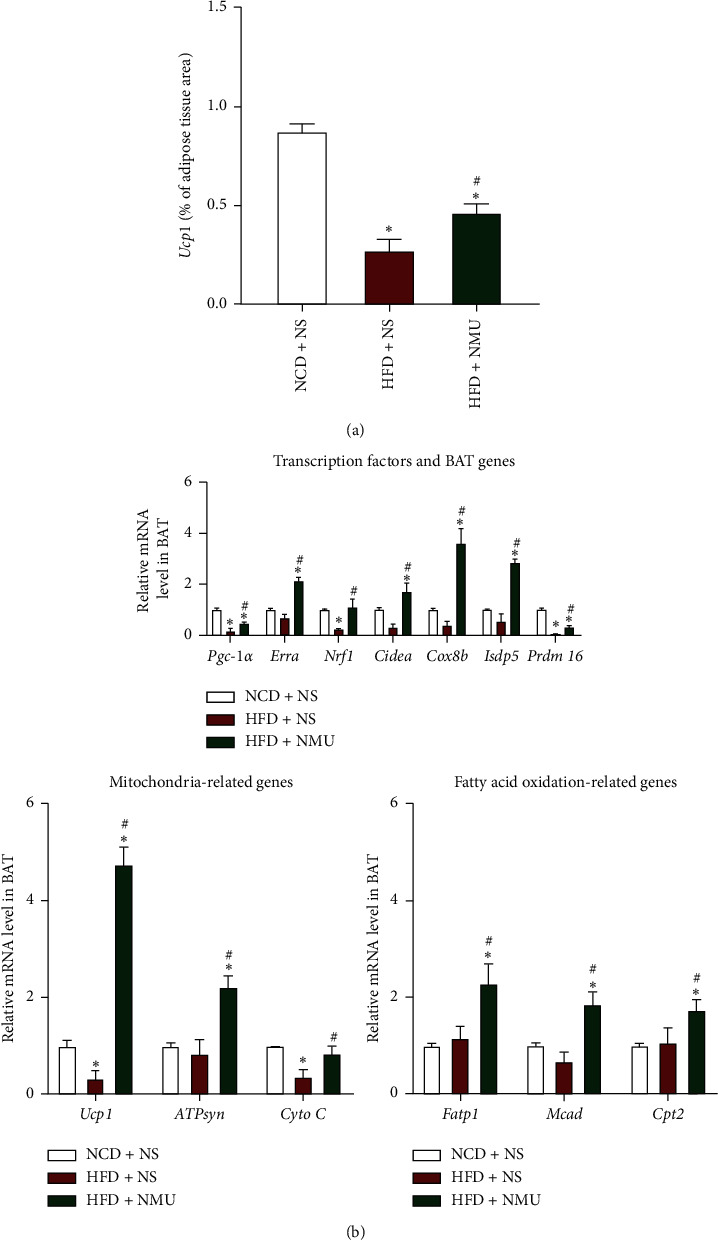
NMU stimulates the expression of thermogenic-related genes in BAT. (a) Quantification of *Ucp1* stain of BAT (*n* = 3). (b) Gene expression profile in BAT (*n* = 5–6). ^*∗*^*p* < 0.05 (compared to the NCD + NS group). ^#^*p* < 0.05 (compared to the HFD + NS group).

In order to determine whether NMU could increase BAT activity in DIO mice, we performed cold-stimulated micro-PET/CT on mice of all 3 groups. BAT signal was clearly observed in the control (NCD + NS) group (white circle) but was scarcely present in DIO mice treated with NS. NMU administration activated interscapular BAT as is shown in PET/CT images (Figure 2(a)). H&E staining showed expanded brown adipocytes with larger lipid droplets in DIO mice, which were reversed in the NMU-treated group (Figures 2(b) and 2(c)). Consistent with phenotypical and morphological changes, immunohistochemistry showed markedly elevated *Ucp1* staining in NMU-treated mice (Figure 3(a)). NMU also activated a network of genes that participate in energy dissipating and thermogenesis according to qPCR. The expressions of transcription factors, including *Pgc-1α*, *Errα*, and *Nrf1*, were significantly up‐regulated in NMU-treated DIO mice. NMU also potently increased the expression of several classical BAT markers, such as *Ucp1*, *Cidea*, *Cox8b*, and *Isdp5*. Components of the mitochondrial electron transport chain and genes related to fatty acid oxidation were also activated (Figure 3(b)). Collectively, our data demonstrates that NMU could restore BAT morphology and function in vivo.

### 3.3. NMU Promotes sWAT Beiging

**Figure 4:**
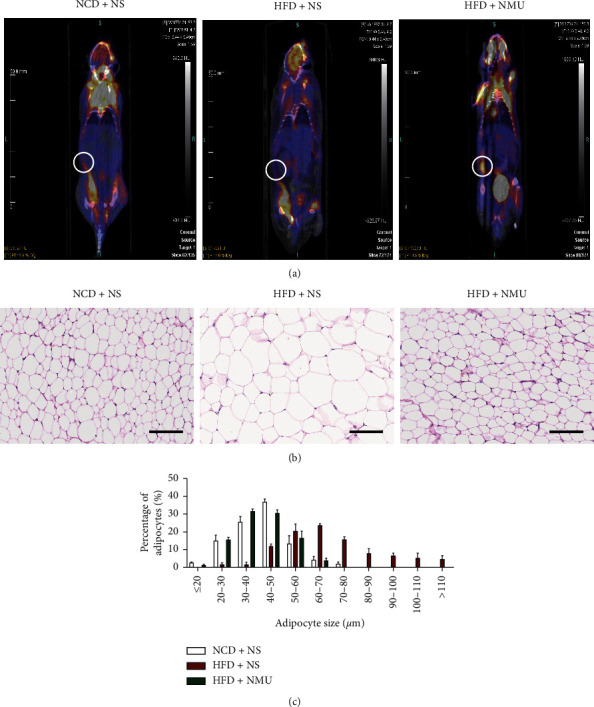
NMU induces brown-like functional and morphological changes in WAT in DIO mice. (a) Coronal view of micro-PET/CT image after cold stimulation and 18F-FDG injection. (b) Representative H&E staining of sWAT. Scale bar, 100 *μ*m. (c) Quantification of adipocytes of different diameters in sWAT (*n* = 3). ^*∗*^*p* < 0.05 (compared to the NCD + NS group). ^#^*p* < 0.05 (compared to the HFD + NS group).

**Figure 5:**
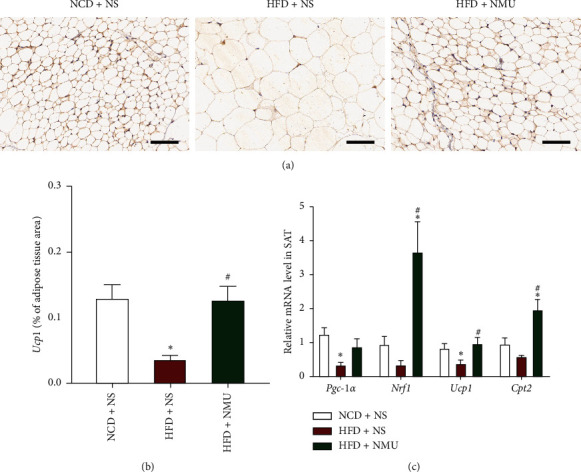
NMU activates thermogenic-related gene profile in sWAT. (a) Representative of immunohistochemical images for *Ucp1* (brown stain) of inguinal sWAT. Scale bar, 100 *μ*m. (b) Quantification of *Ucp1* stain of sWAT (*n* = 3). (c) Thermogenic-related gene expression profile in sWAT (*n* = 5–6). ^*∗*^*p* < 0.05 (compared to the NCD + NS group). ^#^*p* < 0.05 (compared to the HFD + NS group).

We next evaluated indices related to WAT beiging in the 3 groups. Positive signal of sWAT was detected in the inguinal area by PET/CT. The PET/CT signal for inguinal sWAT was negative in DIO mice injected with NS, while after NMU administration, the signal was detected to be higher compared to the other groups (Figure 4(a)). According to H&E staining of paraffin sections, the apparent adipocyte hypertrophy in DIO mice was ameliorated with NMU treatment (Figures 4(b) and 4(c)). Immunohistochemistry exhibited decreased *Ucp1* expression in DIO mice, which was up‐regulated by NMU (Figure 5(a) and 5(b)). The expression of several thermogenic-related genes—*Nrf1*, *Ucp1*, and *Cpt2*—were increased by NMU (Figure 4(c)), indicating sWAT beiging induced by subcutaneous NMU treatment.

### 3.4. NMU Stimulates ILC2s in sWAT

**Figure 6:**
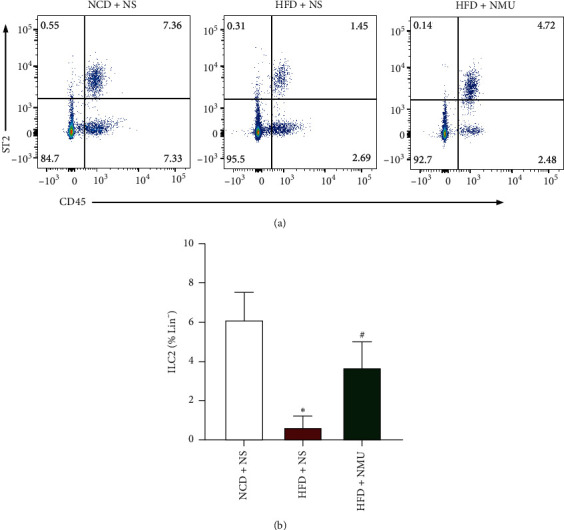
NMU increases ILC2 percentage in WAT in vivo. (a) Representative image of flow cytometry analysis showing the percentage of ILC2s in Lin-cells from mice SVF. (b) Quantification of the percentage of ILC2s in Lin- SVF cells from mice SVF (*n* = 3−4). ^*∗*^*p* < 0.05 (compared to the NCD + NS group). ^#^*p* < 0.05 (compared to the HFD + NS group).

SVF of sWAT was extracted and then analyzed by flow cytometry to examine the changes in ILC2 proportions in different groups. ILC2s were gated on Lin^−^CD45^+^ST2^+^ (Supplementary Figure 1(d)). The percentage of ILC2s in Lin^−^ cells is significantly decreased in DIO mice, while the data suggest that in vivo administration of NMU activated ILC2s in sWAT (Figures 6(a) and 6(b)).

## 4. Discussion

Whether NMU enhances thermogenesis in AT and subsequently improves glucose metabolism remain unclear. In this study, we proved that peripherally administered NMU ameliorated impaired glucose tolerance, activated BAT, and promoted WAT beiging. The changes in WAT may be related to ILC2s stimulation.

Since the discovery that NMU modulates energy homeostasis in vivo, studies have been conducted to evaluate the effects of NMU application for the treatment of obesity and diabetes [21]. NMU, NMU analogs, or NMU receptor agonists exerted glucose-lowering effects when applied to rodents via intracerebroventricular or peripheral injections [16, 21–23]. In our study, the DIO mice were treated with subcutaneously injected NMU. In accordance with previous reports [16], chronic subcutaneous administration of NMU at 8 *μ*g/day did not influence body weight, food intake, or fasting blood glucose (Supplementary Figures 1(a)–1(c)). Unexpectedly, glucose tolerance was markedly improved (Figures 1(a) and 1(b)). It was suggested that the main central effects of NMU were anorexia and weight loss [15, 24], indicating that the improved glucose tolerance in our study was not due to central but direct peripheral effect of NMU.

Previous reports have also identified NMU as a potential thermogenic drug. Intracerebroventricular injection of NMU increased the back surface temperature [15] and core temperature [25] in rodents, while the effect was absent in *Nmur2*-deficient mice [25]. The thermogenic capacity of NMU has been linked to increased BAT activity due to enhanced *Ucp1* expression in BAT after NMU administration [15]. Consistent with previous reports, NMU activated BAT and increased BAT thermogenesis in vivo (Figures 2 and 3). Furthermore, we demonstrated that NMU promoted sWAT beiging, characterized by BAT-like morphological, functional, and genetic alterations in sWAT (Figures 4 and 5). To our knowledge, we demonstrate for the first time that chronic peripheral administration of NMU could promote WAT beiging, which may be implicated in the improvement of glucose tolerance. In the obese population, while WAT depots expand, the percentage of adults with detectable BAT decreased in overweight and obesity [26]. Promoting BAT activation and WAT beiging represent attractive therapeutic strategies in combating obesity and insulin resistance [9, 27]. In adult humans, BAT is a rather vestigial tissue. Less than 10% of adults had positive scans of BAT by 18F-FDG PET/CT. The amount of BAT further diminished with aging and obesity [26, 28]. Thus, the promotion of WAT beiging may provide more promising therapeutic perspectives among diabetic patients who tend to be obese and aged [9].

Recently, it has been pointed out that immune cells within WAT communicate with local nerve fibers to regulate WAT beiging [29]. ILC2s, identified as non-B, non-T lymphocytes that produce type 2 cytokines, were recognized as the upstream components of the axis of ILC2s, eosinophils, and macrophages, which was involved in the regulation of WAT beiging capacity, local immune homeostasis, and AT health [30–32]. NMU was shown to be expressed by cholinergic neurons in the intestine [11, 33]. Intraperitoneal or intranasal-administered NMU activated ILC2s in the small intestine lamina propria and lung [18, 34, 35]. We employed the dosage that was suggested to stimulate ILC2s in vivo [18], with which NMU potently activated ILC2s in sWAT (Figure 6), providing a new perspective in promoting WAT beiging.

In conclusion, we reveal the impact of NMU on glucose metabolism and thermogenesis. Our data also indicate a potential role of the neuro‐immune regulation mediated by NMU and ILC2s in promoting WAT beiging. These findings demonstrate the beneficial metabolic effects of peripherally administered NMU, identifying NMU as a potential drug to enhance thermogenesis and improve glucose tolerance.

## Data Availability

All data generated or analyzed during this study are included in this article and its supplementary information files.

## Supplementary Materials

Supplementary Materials Supplementary Figure 1 Chronic subcutaneous injection of NMU did not impact body weight (a), (*n* = 10–12), food intake (b), (*n* = 3) or FBG (c), (*n* = 5-6). (d) Gating strategy for ILC2s in flow cytometric analysis. Lin: CD3, CD5, TCR*α*/*β*, CD19, CD56, Fc*ε*RI*α*, CD11c, CD11b, CD16. ^*∗*^*p* < 0.05 (compared to NCD + NS group). ^#^*p* < 0.05 (compared to HFD + NS group). Supplementary Table 1 Primers Used for Quantitative real-time PCR analysis.
