# Supplementary material for: Peripheral Administration of NMU Promotes White Adipose Tissue Beiging and Improves Glucose Tolerance
Source: Int J Endocrinol. 2021 Aug 2;2021:6142096. doi: 10.1155/2021/6142096 (PMC8373479; doi:10.1155/2021/6142096)
Supplement: Supplementary Materials — Supplementary Figure 1 Chronic subcutaneous injection of NMU did not impact body weight (a), (n = 10–12), food intake (b), (n = 3) or FBG (c), (n = 5-6). (d) Gating strategy for ILC2s in flow cytometric analysis. Lin: CD3, CD5, TCRα/β, CD19, CD56, FcεRIα, CD11c, CD11b, CD16. ∗p < 0.05 (compared to NCD + NS group). #p < 0.05 (compared to HFD + NS group). Supplementary Table 1 Primers Used for Quantitative real-time PCR analysis. [file 6142096.f1.docx]

**Supplementary Fig. 1** Chronic subcutaneous injection of NMU did not impact body weight (A), food intake (B) or FBG (C). (D) Gating strategy for ILC2s in flow cytometric analysis. Lin: CD3, CD5, TCRα/β, CD19, CD56, FcεRIα, CD11c, CD11b, CD16. The data in (A) and (C) represent the mean ± SEM from at least 3 independent experiments. * p<0.05 (compared to NCD+NS group). # p<0.05 (compared to HFD+NS group).

**Supplementary Table 1** Primers Used for Quantitative real-time PCR analysis.


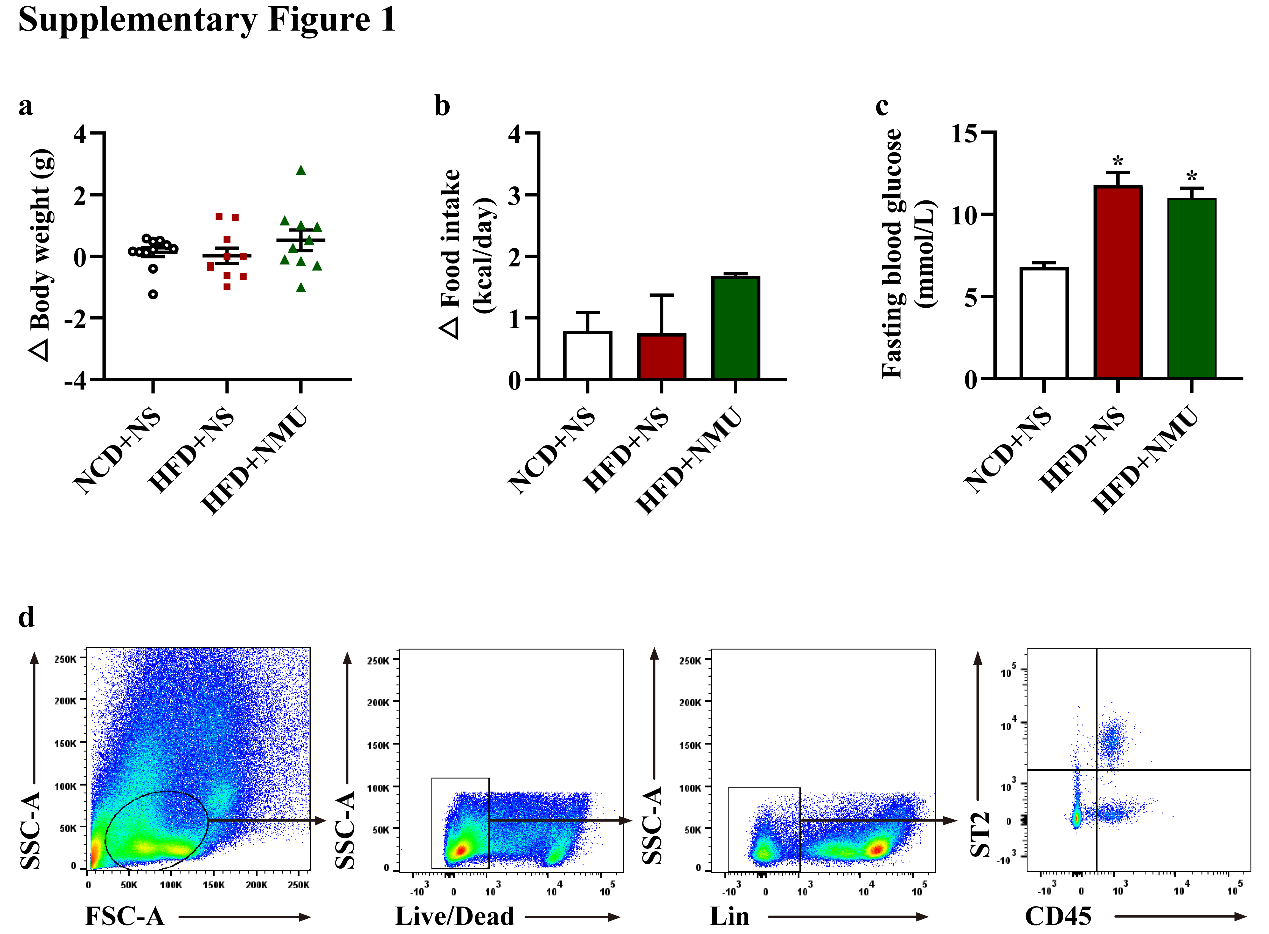


| **Gene name** | **Forward primer sequence (5’-3’)** | **Reverse primer sequence (5’-3’)** |
| --- | --- | --- |
| ***Pgc-1α*** | TATGGAGTGACATAGAGTGTGCT | CCACTTCAATCCACCCAGAAAG |
| ***Ucp1*** | AGGCTTCCAGTACCATTAGGT | CTGAGTGAGGCAAAGCTGATTT |
| ***Cidea*** | TGCTCTTCTGTATCGCCCAGT | GCCGTGTTAAGGAATCTGCTG |
| ***Prdm16*** | CCACCAGCGAGGACTTCAC | GGAGGACTCTCGTAGCTCGAA |
| ***Mcad*** | CCGAAGAGTTGGCGTATGGG | GGGCTCTGTCACACAGTAAGC |
| ***Errα*** | GACGGCAGAAGTACAAACGG | CAACCAGCAGATGCGACAC |
| ***Nrf1*** | AGCACGGAGTGACCCAAAC | TGTACGTGGCTACATGGACCT |
| ***ATP syn*** | GGTTCATCCTGCCAGAGACTA | AATCCCTCATCGAACTGGACG |
| ***Cyto C*** | CCAAATCTCCACGGTCTGTTC | ATCAGGGTATCCTCTCCCCAG |
| ***Cox8b*** | TGTGGGGATCTCAGCCATAGT | AGTGGGCTAAGACCCATCCTG |
| ***Cpt2*** | CCTGCTCGCTCAGGATAAACA | GTGTCTTCAGAAACCGCACTG |
| ***lsdp5*** | CTTCCTGCCCATGACTGAGG | CAGACGCACAAAGTAGCCC |
| ***Fatp1*** | CGCTTTCTGCGTATCGTCTG | GATGCACGGGATCGTGTCT |
| ***Actin*** | GGCTGTATTCCCCTCCATCG | CCAGTTGGTAACAATGCCATGT |

**Supplementary Table 1** Primers Used for Quantitative real-time PCR analysis
